# Comparing the Real-World and Clinical Trial Bleeding Rates Associated with Oral Anticoagulation Treatment for Atrial Fibrillation

**DOI:** 10.3390/jcm13082277

**Published:** 2024-04-15

**Authors:** Ying Gue, Dan Bloomfield, Debra Freedholm, Gregory Y. H. Lip

**Affiliations:** 1 Liverpool Centre for Cardiovascular Science at University of Liverpool, Liverpool John Moores University and Liverpool Heart & Chest Hospital, Liverpool L14 3PE, UK; 2 The Department of Cardiovascular and Metabolic Medicine, University of Liverpool, Liverpool L69 3BX, UK; 3 Anthos Therapeutics, Cambridge, MA 02142, USA; dan.b@anthostherapeutics.com (D.B.); deb.f@anthostherapeutics.com (D.F.); 4 Department of Clinical Medicine, Aalborg University, 9220 Aalborg, Denmark

**Keywords:** atrial fibrillation, stroke, bleeding, real-world clinical practice

## Abstract

**Background** The prevention of stroke in patients with atrial fibrillation (AF) involves the use of oral anticoagulation, commonly in the form of direct oral anticoagulants (DOACs). However, it comes with an increased risk of bleeding, and therefore, counselling patients on their individual risks is important. Although the majority of patients initiated on DOACs have been represented within the clinical trials, some cohorts are under-represented in whom clinicians cannot practice evidence-based medicine. **Methods** Utilising the pooled clinical trial (CT) data sourced from Medidata Enterprise Data Store, five recent open-label industry-sponsored AF trials were compared with real-world data (RWD) sourced from the HealthVerity™ Marketplace with the occurrence of bleeding events as the primary outcome of interest. **Results** A total of 64,421 patients were included in the analysis, with 3207 patients from the clinical DOAC trials and 61,214 patients from the RWD cohort. Overall, the patients from the RWD cohort had more co-morbidities, were older (72.2 ± 11.9 vs. 65.3 ± 10.7 years old, *p* < 0.001), had higher mean CHA_2_DS_2_VASc (3.98 ± 1.9 vs. 2.87 ± 1.73, *p* < 0.001), and HAD-BLED scores (2.13 ± 1.02 vs. 1/04 ± 0.93, *p* < 0.001) when compared to the trial data. When comparing the incidence of the first major bleed at 12 months post-treatment initiation, rates in the RWD cohort were significantly higher (10.69 vs. 18.97 per 100 person-years). The impact of co-morbidities such as age, CHA_2_DS_2_VASc, and HAD-BLED scores was similar in both cohorts; however, there was an under-representation of older females and more co-morbid patients within the clinical trial cohort. **Conclusions** DOAC-treated patients have a higher bleeding incidence rate in the RWD cohort than in clinical trials. This can be explained by the older patient age group with more complex medical h istories and higher HAS-BLED scores. The under-representation of higher-risk patients and lower proportion of females within clinical trials should be addressed to better translate clinical trial data into real-world clinical practice.

## 1. Introduction

Atrial fibrillation (AF) is the most common cardiac arrhythmia associated with a five-fold increased risk of stroke, accounting for nearly a quarter of ischemic strokes and doubling the odds of death [1,2]. The incidence and prevalence of AF have been growing globally [2,3], and the prevalence of AF has tripled in the last five decades [4], which increases healthcare burden and costs [5]. Indeed, the worldwide prevalence of AF has increased from at least 33.5 million people in 2010 to around 46.3 million people in 2016 [6,7]. In the United States, it is projected that by 2050, between 6 and 16 million people will have AF [8]. 

Over 95% of the cases in the United States are non-valvular atrial fibrillation (NVAF) [9], and oral anticoagulation therapy (such as vitamin K antagonists [VKA] and direct oral anticoagulants [DOACs]) for stroke prevention is recommended except for patients with a low risk of stroke, as indicated by a low CHA_2_DS_2_-VASc score, or have specific contraindications [10,11,12]. While oral anticoagulants are used to prevent thromboembolism, this needs to be balanced against the risks of bleeding [10,11].

Clinical trials (CT) are considered the gold standard for generating clinical evidence and are conducted to establish the safety and efficacy of an intervention relative to the standard of care or placebo. These studies are protocol-based, with regimented treatment patterns on selected homogenous patient populations, and are conducted to understand the intervention’s efficacy. Since CT tends to exclude very old or very young patients or patients with significant co-morbidities, the application of such results may be limited as real-world patients are normally more complex as opposed to the highly selected population studied in a tightly controlled clinical setting [13].

There is increasing interest in using real-world data (RWD) for decision-making, especially from regulators. The real-world studies use data generated in real-life clinical practice, where the patient population may not be as selected as one would expect in the CT setting, and the treatment is not as regimented as in the CT setting, with many instances of treatment non-adherence, discontinuation, and switching [13,14]. In RWD, we also have data from a broad spectrum of AF patients, including those with clinical complexity, multimorbidity, and polypharmacy [15,16,17,18].

Therefore, the main goal of this study was to bridge the gap between CT data and real-world clinical practice by examining population characteristics and assessing bleeding outcomes (overall, major, and clinically relevant minor bleeding) in both settings in patients treated with DOAC therapy.

## 2. Methods

### Overview of Study Design

The overarching goal of this study was to describe and compare the patient population of patients treated with DOAC in the CT and the real-world setting and compare the bleeding rates (overall, major, and clinically relevant minor bleeding) in the two data sources. The CT data cohort was pooled across multiple industry-sponsored clinical trials on patients with AF. Only patients exposed to the DOAC were included in the analysis and followed from treatment start (index date). 

For the real-world data, medical and pharmacy claims were used to identify adult patients with atrial fibrillation treated with DOAC prescription (dabigatran, rivaroxaban, apixaban, and edoxaban). The baseline demographic and clinical characteristics and the bleeding rates of the two patient populations were compared.

## 3. Data Source

### 3.1. Clinical Trial Database

The pooled clinical trial data were sourced from Medidata Enterprise Data Store, comprising more than 23,000 historical clinical trials with 6.9 million patients from approximately 1400 customers in around 100 countries over 20 years [19]. The study database included adult patients enrolled in open-label Phase 3 and 4 studies completed between 2014 and 2019 on patients with atrial fibrillation or NVAF treated with DOACs with complete medical history. CT data were standardized using the study data tabulation Model (SDTM). The SDTM defines a standard structure for human clinical study data tabulations and non-clinical study data tabulations to be submitted as part of a product application to a regulatory authority such as the US FDA. 

### 3.2. Real-World Data

The real-world data were sourced from the HealthVerity™ Marketplace Private Source 20 administrative medical and pharmacy claims database, which included Commercial and Medicare Advantage insurance types from the United States of America (USA).

## 4. Study Population

### 4.1. Clinical Trial Database

Only those exposed to the DOAC with available medical history were included in the analysis for the patients in the clinical trial database. Patients were followed from the start of treatment (index date) for a maximum of 12 months. No minimum follow-up period was required for patients to be included in this analysis. No additional inclusion and exclusion criteria were imposed.

### 4.2. Real-World Data

For the patients in the real-world database, the patient identification period was between 1 January 2015 and 30 September 2019, and all the patient-level information from 1 January 2014 and 31 December 2019 was used in the analysis (study period). 

Adult patients (≥18 years) were required to have ≥1 claim(s) with the diagnosis of AF and ≥two prescriptions (on different days) of DOAC (dabigatran, rivaroxaban, apixaban, and edoxaban) during the patient identification period. The index date for the real-world data patients was defined as the date of the first-observed DOAC prescription (dabigatran, rivaroxaban, apixaban, and edoxaban) during the patient identification period. All patients were required to have at least 12 months of continuous eligibility before the index date (baseline period) and were followed for a maximum of 12 months, although no minimum follow-up was imposed.

## 5. Outcomes 

The primary outcome of interest was the occurrence of bleeding events. Major bleed was defined as gastrointestinal bleeding (major GI bleeding included MedDRA preferred terms and ICD and procedure codes associated with GI haemorrhage events [i.e., upper gastrointestinal haemorrhage, gastric ulcer haemorrhage, etc.] intracranial hemorrhage, and other major bleeding (i.e., traumatic hemorrhage, hemorrhage of any major organ, etc.); minor bleed was defined as bleeds classified as non-major (i.e., epistasis, gingival bleeding, etc.), and any bleed was defined as major or minor bleeding [20,21,22,23]. If the first bleeding day had multiple types of bleeding, the cause of bleeding was assigned hierarchically. Priority was given to intracranial bleeding, followed by gastrointestinal bleeding and other major bleeding. In addition, in the RWD, intracranial bleeding was defined as intracranial bleeding with or without codes for hemorrhagic stroke.

## 6. Data Analysis and Statistical Methods

### Statistical Analysis

Baseline characteristics were compared for patients in the RWD and CT database using the chi-squared, Student’s *t*-test for independent groups, and Mann–Whitney U test as appropriate. Incidence rates in the 12-month follow-up were estimated and presented per 100 person-years for both cohorts and by subgroup (age, gender, and HAS-BLED score [24]). Kaplan–Meier curves were used to estimate the time to the first major bleed during the 12-month post-index follow-up period for both cohorts and by the HAS-BLED score. Log-rank tests were used to test differences in intragroup stratifications within each data source. A conventional alpha of 0.05 and a two-tailed level of significance were used for statistical significance without correction for multiple analyses. All statistical analyses were performed using R version 4.0.2.

## 7. Results

**Figure 1:**
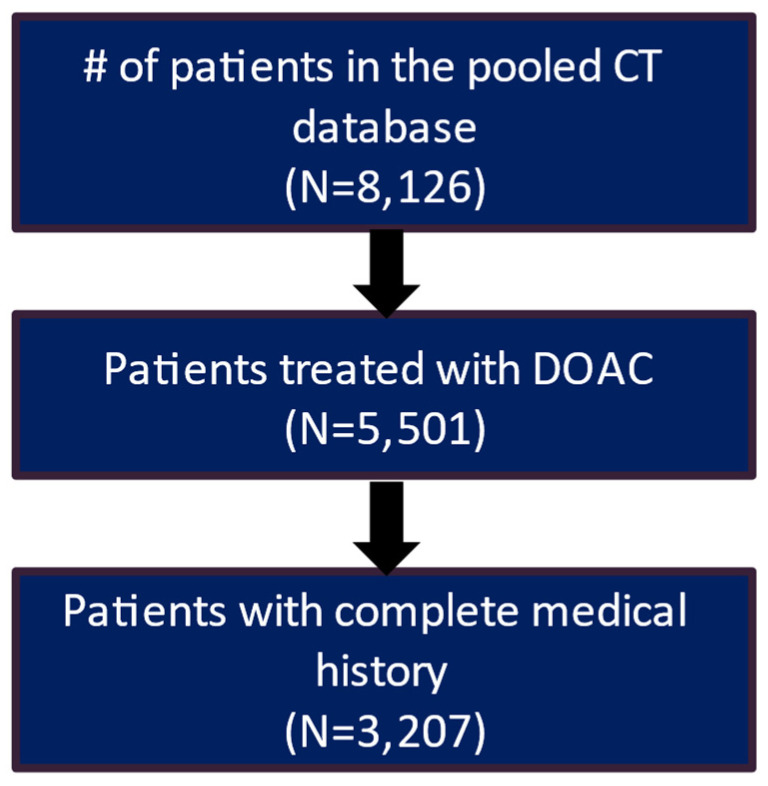
Patient attrition table for CT patients.

**Figure 2:**
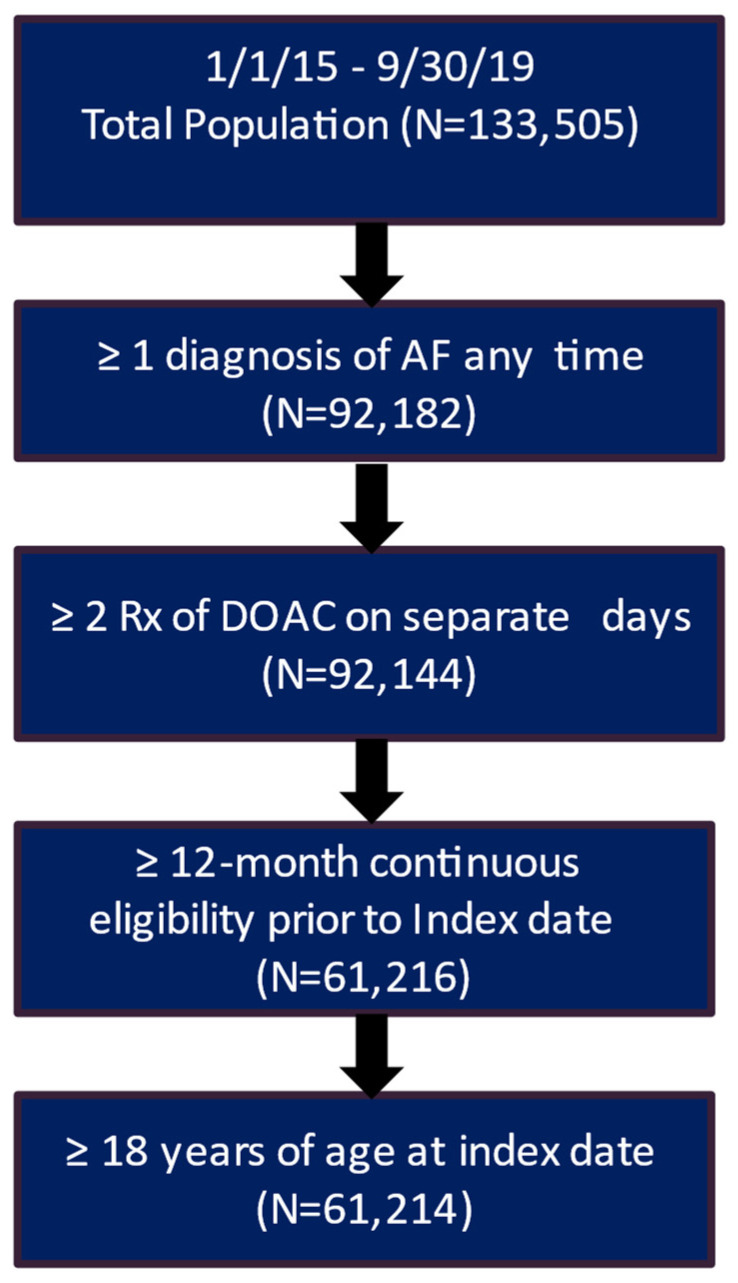
Patient attrition table for RWD patients. AF, atrial fibrillation; DOAC, direct oral anticoagulants.

After applying the inclusion and exclusion criteria, the study included 3207 patients in the clinical trial database treated with DOAC and had a complete medical history. Of these patients, 2217 (69.1%) had a recorded stroke and bleeding risk score (CHA_2_DS_2_-VASc score and HAS-BLED score) (Figure 1). The real-world data included 61,214 eligible patients who had a diagnosis of AF and initiated DOAC treatment during the patient identification period and had more than 12 months of continuous eligibility prior to the start of DOAC treatment (Figure 2). 

## 8. Patient Characteristics

The patients’ baseline demographic and clinical characteristics in the CT database and the RWD are presented in Table 1. Relative to the RWD, in the CT database, the patients were younger (CT vs. RWD: Mean ± SD: 65.3 ± 10.7 vs. 72.2 ± 11.9; *p*-value < 0.001), predominantly male (71% vs. 53%; *p*-value < 0.001), and have a significantly lower proportion of patients had a history of stroke/systemic embolism (SSE) (4.7% vs. 6.8%; *p*-value = 0.012) and other co-morbidities. Additionally, patients in the CT database with similar CHA_2_DS_2_-VASc scores and HAS-BLED scores had a lower risk of stroke (2.9 ± 1.7 vs. 4.0 ± 1.9; *p*-value < 0.001) and bleeding than the RWD patients.

## 9. Incidence Rate of First Bleeding

The number/percentage of bleeding events and the incidence rate of first bleeding during the 12-month follow-up are presented in Table 2. During the 12-month follow-up, patients in the CT database had a lower percentage of patients with major (CT vs. RWD: 3.4% vs. 14.5%), minor (7.3% vs. 33.4%), or any bleeds (11.6% vs. 37.1%). Relative to the RWD, patients in the CT database had a lower incidence of major bleeding (gastrointestinal bleeding, intracranial bleeding, or other major bleeding) events during the 12-month follow-up (CT vs. RWD: 10.69 vs. 18.97 per 100 PY). Intracranial bleeding was similar in both cohorts (0.77 vs. 0.76 per 100 PY), while gastrointestinal bleeding (3.79 vs. 7.61 per 100 PY) and other major bleeding (6.1 vs. 10.6 per 100 PY) were lower in the CT patients. Minor bleed events (30.58 vs. 51.55 per 100 PY) and any bleeding events (40.32 vs. 59.30 per 100 PY) were also lower in the CT database relative to the RWD. 

The major bleeding rates were further evaluated by gender (male vs. female), age categories (18–64, 65–74, 75–78, and 79+ years), and categories by bleeding risk (HAS-BLED) (score of 0, 1, 2, and 3+) (Table 3). Both male and female patients in the CT database had lower major bleeding rates relative to the RWD patients. Across all the age groups, the major bleeding rates were lower for the CT patients relative to the RWD patients. Similarly, across all the HAS-BLED categories, the major bleeding rates were lower for the CT patients relative to the RWD patients.

## 10. Time-to-Bleeding Analysis

**Figure 3:**
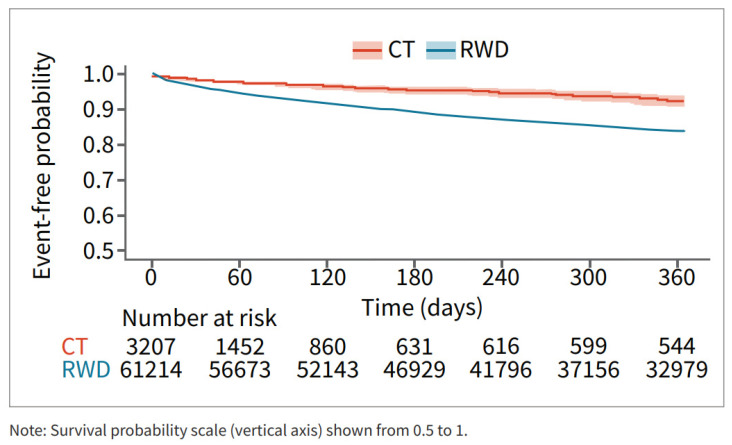
Major bleed risk 12-month follow-up CT and RWD.

Survival analysis showed that patients in the RWD cohort had a higher risk of major bleeding during the 12 months post-DOAC treatment compared to the CT cohort (Figure 3).

**Figure 4:**
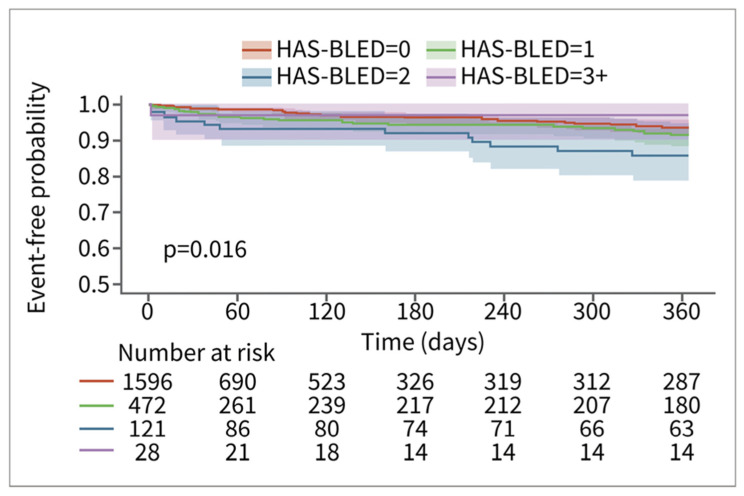
Major bleed risk 12-month follow-up for patients in CT database, stratified via HAS-BLED score.

**Figure 5:**
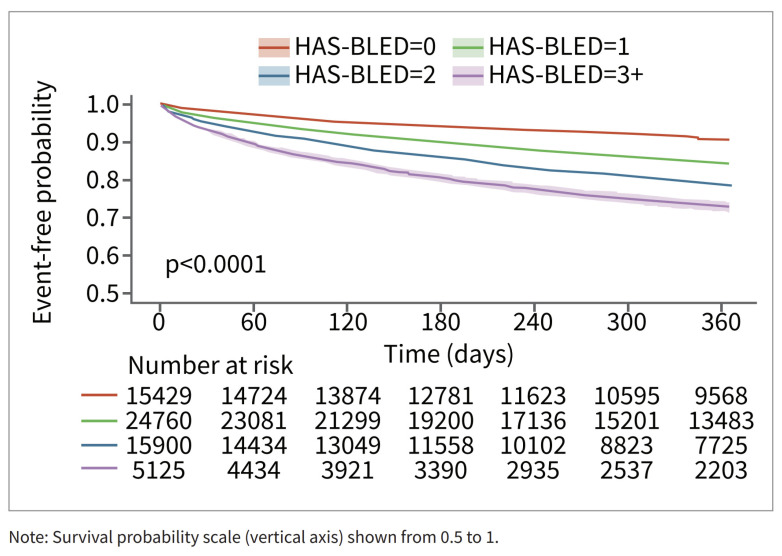
Major bleed risk 12-month follow-up for patients in RWD, stratified via HAS-BLED Score.

Among patients in the RWD, patients with higher HAS-BLED scores had a higher risk of bleeding. The relationship is less pronounced for the patients in the CT database. This may be in part due to smaller sample sizes and consequent counterintuitive results, such as the HAS-BLED > 3 group having the lowest risk of bleeding (*n* = 28) (Figure 4). The relationship between time to major bleeding and HAS-BLED score was more apparent among patients in the RWD, with patients with higher HAS-BLED scores showing a shorter interval of bleeding (Figure 5). Patients in the CT database seem to have a longer time to major bleeding for each HAS-BLED category with sufficient sample size. 

## 11. Discussion

Our study found that relative to the AF patients receiving DOAC in the real world, the patients in the clinical trials assigned to the DOAC arm were younger with lower HAS-BLED and CHA_2_DS_2_-VASc scores prior to initiating DOAC treatment. Second, a lower proportion of patients in the clinical trials had selected co-morbidities, including congestive heart failure, renal disease, coronary artery disease, hypertension, diabetes mellitus, and peripheral arterial disease. Third, while comparing the bleeding outcomes, our study found that patients in the CT database had a numerically lower percentage and incidence of bleeding events and lower risk of major bleeds during the 12-month post-index follow-up period. These differences could be explained by the higher proportion of patients with multiple risk factors within the RWD group.

The HAS-BLED score is considered a well-validated predictor for bleeding among AF patients, in addition to other risk scores such as HEMORR_2_HAGES and ATRIA risk scores [25,26,27,28]. In our study, 93% of patients in the CT database had HAS-BLED ≤ 1, while only 63% of patients in the real-world database had HAS-BLED ≤ 1. This indicates an intrinsic difference in the population included in the two study groups. Apart from the risk factors accounted for in the HAS-BLED score, other risk factors also played a role in the higher incidence of major bleeding. Recent studies evaluating clinical factors and predictors of major bleeding among AF patients treated with VKA or DOAC in the real-world setting found that the history of liver disease, age ≥ 75, antiplatelet use, cardiomyopathy, peripheral arterial disease, and COPD were the most important clinical factors/predictors for major bleeding [29,30,31]. In addition, kidney disease was independently associated with a higher risk for bleeding among AF patients treated with DOAC or VKA [32,33,34]. Indeed, an increase in co-morbidity is associated with an increased risk of bleeding [35].

In our study, nearly 30% of the patients in the real-world database were aged ≥79 years, while only 8% were in the CT database. Proportionally, the RWD data had six times more patients with renal disease (CT vs. RWD: 5% vs. 30%), five times more with peripheral arterial disease (3% vs. 15%), and 1.5 times more patients with diabetes (23% vs. 37%) than in the CT database. Therefore, the differences in patient population are likely to significantly contribute to the observed differences in bleeding risks between the patient populations from two different sources. 

Although more frequent off-label dose adjustments in RWD when compared to CT may have contributed to an increase in event rates, an analysis of apixaban-treated patients showed that event rates in clinical practice compared to CT were consistently higher, irrespective of dosing [36]. This indicates that differences in patient characteristics are additional important contributors and further support our analyses even though dose-related analyses were not performed. 

This highlights the limitations of CT as the strict recruitment criteria would ensure that patients with multiple co-morbidities, i.e., high risk, would be excluded from these trials. With the ageing population, the risk of clinical events increases with implications for oral anticoagulant use. Also, elderly AF patients who are presenting to clinicians are more likely to be multi-morbid, making it more difficult to apply data from CT to the management of these increasingly complex patients.

### 11.1. Clinical Trial Settings and Outcome Definition

Patients in the CT database are monitored more closely than in the real world; therefore, they may have been able to avert bleeding events that patients in the real world could not. This may have artificially reduced the incidence of major bleeding in the CT group. 

Another explanation for the differences may be the way the two data sources defined the bleeding outcomes. The CT database used MedDRA PT terms to identify the bleeding events. The investigators prespecify and review these codes before being entered into the database. In addition, many clinical trials have the Clinical Endpoint Committee (CEC), especially for trials conducted in multiple geographies, which adjudicates the clinical outcomes and are, therefore, likely to be accurately captured [37,38]. On the other hand, the claims databases were originally fashioned for reimbursement purposes; therefore, the reporting of events is strongly influenced by whether an event is reimbursable or not and, if reimbursable, its reimbursement rate. This may affect the rate and congruency of the bleeding events between the CT and real-world databases. 

### 11.2. Limitations

There are several limitations to our study. Being a retrospective study, the data could be at risk of bias, particularly with RWD, as stated above with respect to reimbursements. The lack of adjudication of the outcomes would also reduce the reliability of the RWD.

Secondly, apart from differences in clinical parameters, differences between baseline populations could partly contribute to the differences in bleeding events. CT data included patients from over 100 countries, whereas RWD are only from the US. Previous studies have shown ethnic and racial differences in bleeding complications associated with DOACs [38,39], and hence, the population/ethnic differences could have impacted the results seen in our study, given ethic differences in stroke and bleeing rates [40,41,42].

Lastly, the lack of information on concomitant pharmacological therapy, such as the use of antiplatelets, is an important limitation, as this would have an impact on bleeding outcomes. Similarly, as there is no minimal time on DOAC required or information regarding therapy prior to DOAC, the possible lower adherence and compliance in the RWD group might have an impact on outcomes. 

## 12. Conclusions

Despite other differences between the real-world and CT data, most of the differences in the bleeding rates between the two data sources are driven by the differences in the patient population, and the CT data underestimates the burden of bleeding in real-world clinical practice due to a lower representation of elderly and high-risk patients compared to RWD. Evaluating CT data and RWD provides an opportunity to improve future CT design and better align with real-world practice by identifying populations with less representation and subgroups that may influence outcomes.

## Figures and Tables

**Table 1:** Patient characteristics.

	Clinical Trial Database	Real-World Database	*p*-Value
	**N = 3207**	**N = 61,214**	
Age [Mean, (SD)]	65.3 (10.7)	72.2 (11.9)	<0.001
18–64 years old [%]	48.7	28.2	<0.001
65–74 years old [%]	34.2	27.7	
75–78 years old [%]	9.3	15.4	
79+ years old [%]	7.8	28.7	
Male [%]	70.5	53.0	<0.001
Stroke/Systemic embolism [%]	4.7	6.8	0.012
History of Congestive Heart Failure [%]	33.1	35.3	<0.001
History of Renal Disease [%]	5.0	30.3	<0.001
History of Coronary Artery Disease [%]	18.0	44.0	<0.001
History of Hypertension [%]	75.7	86.6	<0.001
History of Diabetes Mellitus [%]	22.7	36.7	<0.001
History of Peripheral Arterial Disease [%]	3.3	14.7	<0.001
	**N = 2217**	**N = 61,214**	
HAS-BLED [Mean, (SD)]	1.04 (0.93)	2.13 (1.02)	<0.001
HAS-BLED Score 0 [%]	72.0	25.2	<0.001
HAS-BLED Score 1 [%]	21.3	40.4	
HAS-BLED Score 2 [%]	5.5	26.0	
HAS-BLED Score 3+ [%]	1.3	8.4	
CHA_2_DS_2_-VASc score [Mean, (SD)]	2.87 (1.73)	3.98 (1.9)	<0.001
CHA_2_DS_2_-VASc score 0 [%]	22.2	10.5	<0.001
CHA_2_DS_2_-VASc score 1 [%]	23.6	13.1	
CHA_2_DS_2_-VASc score 2 [%]	20.2	16.8	
CHA_2_DS_2_-VASc score 3 [%]	16.3	19.6	
CHA_2_DS_2_-VASc score 4+ [%]	17.7	40.0	

**Table 2:** Bleeding events during the 12-month follow-up period.

	Clinical Trial Database (N = 3207)		Real-World Data (N = 61,214)	
	Number of Events (n, %)	Incidence Rate (per 100 Person-Years (PY)	Number of Events (n, %)	Incidence Rate (per 100 Person-Years (PY)
Any Bleeding, event	372 (11.6%)	40.32	22,691 (37.1%)	59.30
Minor Bleeding	289 (7.3%)	30.58	20,437 (33.4%)	51.55
Major ^a^ Bleeding	110 (3.4%)	10.69	8856 (14.5%)	18.97
Gastrointestinal Bleeding	39 (35.5%)	3.79	3551 (40.1%)	7.61
Intracranial Bleeding ^b^	8 (7.3%)	0.77	357 (4.0%)	0.76
Other major Bleeding ^c^	63 (57.3%)	6.13	4948 (57.3%)	10.60

^a^ Same-day multiple bleeds were categorized in the following priority: 1. intracranial bleeding; 2. GI bleeding; 3. other major bleeding. ^b^ In the RWD, it was defined as having intracranial bleeding with or without codes for hemorrhagic stroke. ^c^ RWD hemorrhagic stroke was grouped under other major bleeding.

**Table 3:** Major bleeding rates during the 12-month follow-up stratified by gender, age, and HAS-BLED.

	Clinical Trial Database	Real-World Data
	N = 3207	N = 61,214
Sex, event per 100 PY (%)		
Female	10.81	22.31
Male	10.64	16.13
Age, event per 100 PY (%)		
18–64	7.77	12.99
65–74	11.58	18.64
75–78	15.73	22.76
79+	14.55	24.67
HAS-BLED scores, event per 100 PY (%)	N = 2217	N = 61,214
0	9.14	10.07
1	11.32	17.88
2	17.9	25.52
3+	6.50	35.60

## Data Availability

The real-world data underlying this article were provided by HealthVerity under licence/by permission. Data will be shared on request to the corresponding author with permission of HealthVerity. The clinical trials data underlying this article cannot be shared publicly due to privacy of individuals that participated in the study. The data will be shared on reasonable request to the corresponding author.

## References

[1] Marini C., De Santis F., Sacco S., Russo T., Olivieri L., Totaro R., Carolei A. (2005). Contribution of atrial fibrillation to incidence and outcome of ischemic stroke: Results from a population-based study. Stroke.

[2] Zhang J., Johnsen S.P., Guo Y., Lip G.Y.H. (2021). Epidemiology of Atrial Fibrillation: Geographic/Ecological Risk Factors, Age, Sex, Genetics. Card. Electrophysiol. Clin..

[3] Kornej J., Börschel C.S., Benjamin E.J., Schnabel R.B. (2020). Epidemiology of Atrial Fibrillation in the 21st Century: Novel Methods and New Insights. Circ. Res..

[4] Schnabel R.B., Yin X., Gona P., Larson M.G., Beiser A.S., McManus D.D., Newton-Cheh C., Lubitz S.A., Magnani J.W., Ellinor P.T. (2015). 50 year trends in atrial fibrillation prevalence, incidence, risk factors, and mortality in the Framingham Heart Study: A cohort study. Lancet.

[5] Vîjan A.E., Daha I.C., Delcea C., Dan G.-A. (2021). Determinants of Prolonged Length of Hospital Stay of Patients with Atrial Fibrillation. J. Clin. Med..

[6] Benjamin E.J., Muntner P., Alonso A., Bittencourt M.S., Callaway C.W., Carson A.P., Chamberlain A.M., Chang A.R., Cheng S., Das S.R. (2019). Heart Disease and Stroke Statistics-2019 Update: A Report from the American Heart Association. Circulation.

[7] Chugh S.S., Havmoeller R., Narayanan K., Singh D., Rienstra M., Benjamin E.J., Gillum R.F., Kim Y.H., McAnulty J.H., Zheng Z.J. (2014). Worldwide epidemiology of atrial fibrillation: A Global Burden of Disease 2010 Study. Circulation.

[8] Miyasaka Y., Barnes M.E., Gersh B.J., Cha S.S., Bailey K.R., Abhayaratna W.P., Seward J.B., Tsang T.S. (2006). Secular trends in incidence of atrial fibrillation in Olmsted County, Minnesota, 1980 to 2000, and implications on the projections for future prevalence. Circulation.

[9] Go A.S., Hylek E.M., Phillips K.A., Chang Y., Henault L.E., Selby J.V., Singer D.E. (2001). Prevalence of diagnosed atrial fibrillation in adults: National implications for rhythm management and stroke prevention: The AnTicoagulation and Risk Factors in Atrial Fibrillation (ATRIA) Study. JAMA.

[10] January C.T., Wann L.S., Calkins H., Chen L.Y., Cigarroa J.E., Cleveland J.C., Ellinor P.T., Ezekowitz M.D., Field M.E., Furie K.L. (2019). 2019 AHA/ACC/HRS Focused Update of the 2014 AHA/ACC/HRS Guideline for the Management of Patients with Atrial Fibrillation: A Report of the American College of Cardiology/American Heart Association Task Force on Clinical Practice Guidelines and the Heart Rhythm Society in Collaboration with the Society of Thoracic Surgeons. Circulation.

[11] Gorog D.A., Gue Y.X., Chao T.-F., Fauchier L., Ferreiro J.L., Huber K., Konstantinidis S.V., Lane D.A., Marin F., Oldgren J. (2022). Assessment and Mitigation of Bleeding Risk in Atrial Fibrillation and Venous Thromboembolism: Executive Summary of a European and Asia-Pacific Expert Consensus Paper. Thromb. Haemost..

[12] Chao T.F., Joung B., Takahashi Y., Lim T.W., Choi E.K., Chan Y.H., Guo Y., Sriratanasathavorn C., Oh S., Okumura K. (2022). 2021 Focused Update Consensus Guidelines of the Asia Pacific Heart Rhythm Society on Stroke Prevention in Atrial Fibrillation: Executive Summary. Thromb. Haemost..

[13] Cohen A.T., Goto S., Schreiber K., Torp-Pedersen C. (2015). Why do we need observational studies of everyday patients in the real-life setting?. Eur. Heart J. Suppl..

[14] Fanaroff A.C., Steffel J., Alexander J.H., Lip G.Y.H., Califf R.M., Lopes R.D. (2018). Stroke prevention in atrial fibrillation: Re-defining ‘real-world data’ within the broader data universe. Eur. Heart J..

[15] Zheng Y., Li S., Liu X., Lip G.Y.H., Guo L., Zhu W. (2023). Effect of Oral Anticoagulants in Atrial Fibrillation Patients with Polypharmacy: A Meta-analysis. Thromb. Haemost..

[16] Grymonprez M., Petrovic M., De Backer T.L., Steurbaut S., Lahousse L. (2024). The Impact of Polypharmacy on the Effectiveness and Safety of Non-vitamin K Antagonist Oral Anticoagulants in Patients with Atrial Fibrillation. Thromb. Haemost..

[17] Romiti G.F., Proietti M., Bonini N., Ding W.Y., Boriani G., Huisman M.V., Lip G.Y.H. (2022). Clinical Complexity Domains, Anticoagulation, and Outcomes in Patients with Atrial Fibrillation: A Report from the GLORIA-AF Registry Phase II and III. Thromb. Haemost..

[18] Deitelzweig S., Keshishian A., Kang A., Dhamane A.D., Luo X., Klem C., Rosenblatt L., Mardekian J., Jiang J., Yuce H. (2021). Use of Non-Vitamin K Antagonist Oral Anticoagulants among Patients with Nonvalvular Atrial Fibrillation and Multimorbidity. Adv. Ther..

[19] (2022). The Medidata Enterprise Data Store: The Data Foundation of the Medidata Clinical Cloud^TM^.

[20] Lip G.Y., Nieuwlaat R., Pisters R., Lane D.A., Crijns H.J. (2010). Refining clinical risk stratification for predicting stroke and thromboembolism in atrial fibrillation using a novel risk factor-based approach: The euro heart survey on atrial fibrillation. Chest.

[21] Cunningham A., Stein C.M., Chung C.P., Daugherty J.R., Smalley W.E., Ray W.A. (2011). An automated database case definition for serious bleeding related to oral anticoagulant use. Pharmacoepidemiol. Drug Saf..

[22] Graham D.J., Reichman M.E., Wernecke M., Zhang R., Southworth M.R., Levenson M., Sheu T.C., Mott K., Goulding M.R., Houstoun M. (2015). Cardiovascular, bleeding, and mortality risks in elderly Medicare patients treated with dabigatran or warfarin for nonvalvular atrial fibrillation. Circulation.

[23] Hart R.G., Pearce L.A., Aguilar M.I. (2007). Meta-analysis: Antithrombotic therapy to prevent stroke in patients who have nonvalvular atrial fibrillation. Ann. Intern. Med..

[24] Pisters R., Lane D.A., Nieuwlaat R., de Vos C.B., Crijns H.J., Lip G.Y. (2010). A novel user-friendly score (HAS-BLED) to assess 1-year risk of major bleeding in patients with atrial fibrillation: The Euro Heart Survey. Chest.

[25] Nishimoto Y., Yamashita Y., Morimoto T., Saga S., Amano H., Takase T., Hiramori S., Kim K., Oi M., Akao M. (2020). Validation of the VTE-BLEED score’s long-term performance for major bleeding in patients with venous thromboembolisms: From the COMMAND VTE registry. J. Thromb. Haemost..

[26] Roldán V., Marín F., Manzano-Fernández S., Gallego P., Vílchez J.A., Valdés M., Vicente V., Lip G.Y. (2013). The HAS-BLED score has better prediction accuracy for major bleeding than CHADS2 or CHA2DS2-VASc scores in anticoagulated patients with atrial fibrillation. J. Am. Coll. Cardiol..

[27] Zhu W., He W., Guo L., Wang X., Hong K. (2015). The HAS-BLED Score for Predicting Major Bleeding Risk in Anticoagulated Patients with Atrial Fibrillation: A Systematic Review and Meta-analysis. Clin. Cardiol..

[28] Borre E.D., Goode A., Raitz G., Shah B., Lowenstern A., Chatterjee R., Sharan L., Allen LaPointe N.M., Yapa R., Davis J.K. (2018). Predicting Thromboembolic and Bleeding Event Risk in Patients with Non-Valvular Atrial Fibrillation: A Systematic Review. Thromb. Haemost..

[29] Qazi J.Z., Schnitzer M.E., Côté R., Martel M.-J., Dorais M., Perreault S. (2021). Predicting major bleeding among hospitalized patients using oral anticoagulants for atrial fibrillation after discharge. PLoS ONE.

[30] Sakuma I., Uchiyama S., Atarashi H., Inoue H., Kitazono T., Yamashita T., Shimizu W., Ikeda T., Kamouchi M., Kaikita K. (2019). Clinical risk factors of stroke and major bleeding in patients with non-valvular atrial fibrillation under rivaroxaban: The EXPAND Study sub-analysis. Heart Vessel..

[31] Claxton J.S., MacLehose R.F., Lutsey P.L., Norby F.L., Chen L.Y., O’Neal W.T., Chamberlain A.M., Bengtson L.G.S., Alonso A. (2018). A new model to predict major bleeding in patients with atrial fibrillation using warfarin or direct oral anticoagulants. PLoS ONE.

[32] Bonde A.N., Lip G.Y., Kamper A.L., Fosbøl E.L., Staerk L., Carlson N., Torp-Pedersen C., Gislason G., Olesen J.B. (2016). Renal Function and the Risk of Stroke and Bleeding in Patients with Atrial Fibrillation: An Observational Cohort Study. Stroke.

[33] Becattini C., Giustozzi M., Ranalli M.G., Bogliari G., Cianella F., Verso M., Agnelli G., Vedovati M.C. (2018). Variation of renal function over time is associated with major bleeding in patients treated with direct oral anticoagulants for atrial fibrillation. J. Thromb. Haemost..

[34] Mitsuma W., Matsubara T., Hatada K., Imai S., Saito N., Shimada H., Miyazaki S. (2016). Clinical characteristics of hemodialysis patients with atrial fibrillation: The RAKUEN (Registry of atrial fibrillation in chronic kidney disease under hemodialysis from Niigata) study. J. Cardiol..

[35] Proietti M., Esteve-Pastor M.A., Rivera-Caravaca J.M., Roldán V., Roldán Rabadán I., Muñiz J., Cequier Á., Bertomeu-Martínez V., Badimón L., Anguita M. (2021). Relationship between multimorbidity and outcomes in atrial fibrillation. Exp. Gerontol..

[36] de Vries T.A.C., Hirsh J., Xu K., Mallick I., Bhagirath V.C., Eikelboom J.W., Ginsberg J.S., Kruger P.C., Chan N.C. (2020). Apixaban for Stroke Prevention in Atrial Fibrillation: Why are Event Rates Higher in Clinical Practice than in Randomized Trials?—A Systematic Review. Thromb. Haemost..

[37] Held C. (2019). When do we need clinical endpoint adjudication in clinical trials?. Ups. J. Med. Sci..

[38] Meah M.N., Denvir M.A., Mills N.L., Norrie J., Newby D.E. (2020). Clinical endpoint adjudication. Lancet.

[39] Bang O.Y., Hong K.S., Heo J.H., Koo J., Kwon S.U., Yu K.H., Bae H.J., Lee B.C., Yoon B.W., Kim J.S. (2014). New oral anticoagulants may be particularly useful for asian stroke patients. J. Stroke.

[40] Tamargo J., Kaski J.C., Kimura T., Barton J.C., Yamamoto K., Komiyama M., Drexel H., Lewis B.S., Agewall S., Hasegawa K. (2022). Racial and ethnic differences in pharmacotherapy to prevent coronary artery disease and thrombotic events. Eur. Heart J. Cardiovasc. Pharmacother..

[41] Kang D.S., Yang P.S., Kim D., Jang E., Yu H.T., Kim T.H., Sung J.H., Pak H.N., Lee M.H., Lip G.Y.H. (2024). Racial Differences in Ischemic and Hemorrhagic Stroke: An Ecological Epidemiological Study. Thromb. Haemost..

[42] Kang D.S., Yang P.S., Kim D., Jang E., Yu H.T., Kim T.H., Sung J.H., Pak H.N., Lee M.H., Lip G.Y.H. (2024). Racial Differences in Bleeding Risk: An Ecological Epidemiological Study Comparing Korea and United Kingdom Subjects. Thromb. Haemost..

